# Shared Vision for Improving Outcomes for Serious Fungal Diseases: Report of a Patient, Caregiver, and Clinician Summit

**DOI:** 10.1093/ofid/ofae226

**Published:** 2024-04-26

**Authors:** Rob Purdie, Lisa A Tushla, Jonathan Ferretti, Gonzalo (Kiko) Castro, Ricky Watson, Thomas Davis, Brianna Raborg, Patrick B Mazi, Angela Stroman, Carolynn Thomas Jones, Thomas J Walsh, Tom M Chiller, Peter G Pappas, John Meyer, Andrej Spec

**Affiliations:** Integrita Healthcare Education Foundation/MyCare, Colorado Springs, Colorado, USA; Valley Fever Institute, Kern Medical, Bakersfield, California, USA; Taskforce of AMR Survivors, World Health Organization, Geneva, Switzerland; Integrita Healthcare Education Foundation/MyCare, Colorado Springs, Colorado, USA; Integrita Healthcare Education Foundation/MyCare, Colorado Springs, Colorado, USA; Integrita Healthcare Education Foundation/MyCare, Colorado Springs, Colorado, USA; Integrita Healthcare Education Foundation/MyCare, Colorado Springs, Colorado, USA; Integrita Healthcare Education Foundation/MyCare, Colorado Springs, Colorado, USA; Integrita Healthcare Education Foundation/MyCare, Colorado Springs, Colorado, USA; Integrita Healthcare Education Foundation/MyCare, Colorado Springs, Colorado, USA; Division of Infectious Diseases, Washington University, St. Louis, Missouri, USA; Division of Pulmonary and Critical Care Medicine, Washington University, St. Louis, Missouri, USA; Infectious Diseases Translational Research Unit, Augusta University, Augusta, Georgia, USA; College of Nursing, The Ohio State University, Columbus, Ohio, USA; Mycoses Study Group Education and Research Consortium, Birmingham, Alabama, USA; Center for Innovative Therapeutics and Diagnostics, Richmond, Virginia; Departments of Medicine and Microbiology & Immunology, University of Maryland School of Medicine, Baltimore, Maryland; Mycotic Diseases Branch, Centers for Disease Control and Prevention, Atlanta, Georgia, USA; Mycoses Study Group Education and Research Consortium, Birmingham, Alabama, USA; University of Alabama at Birmingham, Division of Infectious Diseases, Birmingham, Alabama, USA; Integrita Healthcare Education Foundation/MyCare, Colorado Springs, Colorado, USA; Integrita Healthcare Education Foundation/MyCare, Colorado Springs, Colorado, USA; Division of Infectious Diseases, Washington University, St. Louis, Missouri, USA

**Keywords:** disease burden, fungal disease, patient advocacy, patient-important outcomes, fungal diagnostics

## Abstract

**Background:**

Recently, increasing focus on patient input into research and healthcare improvements has fostered expanded patient-centered advocacy efforts. This first pan-fungal disease summit, part of the MYCology Advocacy, Research, & Education effort, brought together patients, caregivers, and mycology experts to better document patient experiences with invasive fungal disease (IFD) and establish priorities for mycology education, advocacy, and research.

**Methods:**

Patients who had suffered from IFD, their caregivers, clinicians, industry representatives, government officials, and patient advocacy professionals were invited. Patients and caregivers shared their stories and struggles with IFD. Breakout sessions separated mycology experts from patients and caregivers for further discussions to identify commonalities and perceived gaps and to formulate recommendations. The 2 groups then reconvened to develop consensus recommendations.

**Results:**

IFD patients and their caregivers shared experiences reflecting the typically lengthy prediagnosis, acute treatment, long-term treatment, and posttreatment recovery stages of IFD. They reported substantial physical, psychological, and financial burdens associated with the IFD experience, particularly related to delayed diagnoses. They reaffirmed a need for coordinated patient-centered education, peer support, and advocacy to document the burden of serious fungal infections. Mycology experts discussed strategies to address gaps in the mycology field, such as insufficient training, inadequate workforce support, and a need to partner more with patient groups.

**Conclusions:**

A summit involving patients with IFD, family caregivers, and mycology experts identified a substantial nonclinical burden of disease associated with IFD. Patients and mycology experts prioritized several goals for education, advocacy, and research to raise awareness of IFD and improve outcomes.

## Graphical Abstract

**Graphical Abstract:**
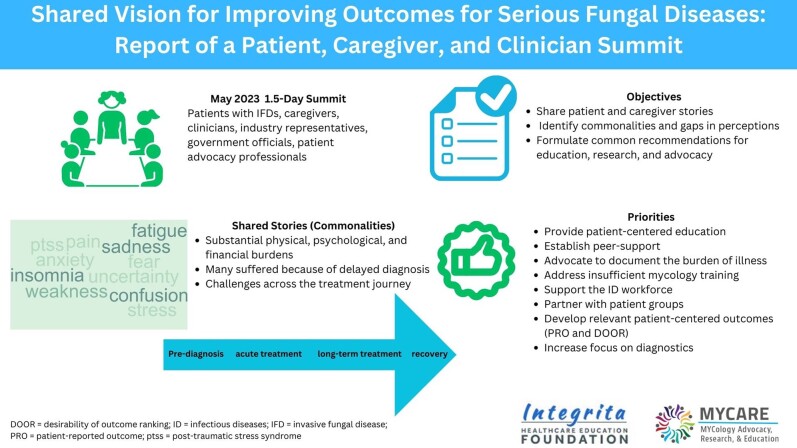

## Teaser

A report from a summit comprised of patients with invasive fungal disease (IFD), family caregivers, and mycology experts identified substantial physical, psychological, and financial burdens associated with the IFD experience. Patient-relevant priorities for mycology education, advocacy, and research were identified.

The increasing burden of serious fungal diseases is a growing public health concern, with an annual incidence of 6.5 million cases worldwide and 2.5 million directly attributable deaths [1]. However, the true disease burden of serious fungal disease is likely underestimated. Indeed, priorities in mycology include defining the public health burden of fungal diseases, understanding the geographic distribution of endemic fungi, and identifying the groups of people at highest risk for invasive fungal disease (IFD) [2]. Antifungal resistance is also a global public health concern [3–6]. The incomplete picture of the fungal disease burden is intertwined with a lack of knowledge among healthcare providers, patients, and the general public, particularly regarding emerging threats.

Some of the most effective disease-awareness efforts are led by or supported by patient-centered nonprofit organizations. Examples include work in amyotrophic lateral sclerosis [7], human immunodeficiency virus/acquired immune deficiency syndrome [8, 9], and cystic fibrosis [10]. In fungal disease advocacy, a global advocacy initiative by the *Global Action for Fungal Infections* [11] aims to improve fungal diagnosis and treatment models for low- to middle-income countries. To capture patient perspectives on treatment experiences, disease outcomes, and unmet needs across medical mycology, the Mycoses Study Group Education & Research Consortium (MSGERC) led the Faces of Fungal Infection video effort, capturing patient stories and clinician discussion across a range of fungal diseases [12]. Some disease-specific advocacy efforts have been advanced in mycology. Patients suffering from coccidioidomycoses (Valley Fever) have been the most successful in advocacy but only on a limited scale [13, 14]. However, a pan-fungal disease patient-led advocacy effort has been lacking until this effort.

The vision for this effort began nearly a decade ago. Key leaders have been involved with patient advocacy initiatives in specialized fungal disease areas (eg, pediatric fungal diseases, mucormycosis, coccidioidomycosis) and in oncology [15–19]. Collectively, patients, clinicians, and advocates recognized the need for a more comprehensive fungal disease patient advocacy effort, given the commonalities of challenges and experiences across medical mycology. The medical community in general as well as the US Food and Drug Administration have emphasized the critical importance of including the patient voice in education, research, and drug development [20, 21]. Inclusion of the patient's voice is the new normal.

This pan-fungal disease advocacy effort was initiated during a collaboration between the MSGERC, Terranova Medica, LLC, Integrita Healthcare Education & Research Foundation, and the Valley Fever Institute through a Centers for Disease Control and Prevention cooperative agreement related to COVID-19-associated IFD. The collaborating individuals established the Mycology Advocacy, Research, & Education (MyCARE) initiative.

The first MyCARE Pan-fungal Disease Patient, Caregiver, and Clinician Summit was held May 19–20, 2023, in Denver, Colorado. Stakeholders came together to hear patient/caregiver stories and experiences and prioritize next steps in education, research, and advocacy for IFD. The summit was held to provide an avenue for patients and caregivers to share their experiences, explore unique patient and caregiver needs, identify the patient-important outcomes, and establish priorities for MyCARE's initial planning efforts. The purpose of this paper is to share the methods and outcomes of the summit.

## METHODS

The day-and-a-half summit included patients who have experienced IFD, their family caregivers, physician researchers, nurses, medical educators, patient advocacy organization members, government officials, and industry representatives. The summit was designed to document the patient and caregiver perspectives on the disease journey and to define unmet needs and goals for education, fungal research, and mycology advocacy.

Patients were chosen to represent a variety of types of IFD through collaboration with physicians who supported the summit. They had been diagnosed with the following IFDs: COVID-19-associated pulmonary aspergillosis, disseminated histoplasmosis, scopulariopsis, cryptococcal meningitis, mucormycosis, and coccidioidomycosis. All patients were male, 1 was a teenager and the rest were working-age adults at the time of diagnosis. Host characteristics included stem cell transplantation, solid organ transplantation, COVID-19, diabetes mellitus, and normal characteristics (for the 2 endemic cases). One patient was Latinx, the rest were White. Although most patients were insured, 1 was without insurance and receiving public assistance at the time of diagnosis. Each participant had a female family caregiver who also attended to share their perspectives. Most patients were invited to the summit by their clinicians, who were also key participants in the summit. MyCARE paid for travel to the summit and provided a stipend. Six clinicians, including 2 advanced practice provider nurses, attended. In addition to the MyCARE staff and MSGERC representatives, representatives from 3 pharmaceutical companies supporting the summit and 1 representative from the Centers for Disease Control and Prevention attended.

The summit began with the patients and caregivers sharing their stories in an open-forum discussion. After this, 2 groups (patient/caregivers and mycology experts/clinicians) broke into separate discussion rooms to brainstorm on educational, research, and advocacy priorities. After each breakout, the groups were brought together to summarize their sessions and to reach consensus recommendations. The attendees also listened to summaries of research priorities from the experts and examples of advocacy efforts from other organizations. The collective discussion forum and breakout sessions were repeated 2 additional times. Before the meeting closed, participants completed an evaluation of the process and priorities. After the meeting, a follow-up survey was sent to the patients and caregivers via e-mail to capture quantitative data about their personal experiences.

The institutional review board of the Washington University School of Medicine deemed the summit and the reporting of outcomes as exempt from review.

## RESULTS

### Patient and Caregiver Stories

Extremes of emotions were present in the stories shared by patients and their caregivers. Fear, exhaustion, and profound sadness peaked during periods of intensive care unit admissions, uncertain diagnoses, worsening symptoms, and when patients were given difficult survival odds. Hope, optimism, and gratitude highlighted periods of diagnostic certainty, eased suffering, treatment successes, and discharges to home. The experiences of these individuals were informative, emotionally profound, and they visibly affected every person in the room. Recognizing the importance of hearing these stories, the group unanimously agreed to extend the session well beyond the allotted time. Any attempt to capture their stories in a few paragraphs here would be a disservice to their experiences. Rather, we will honor their shared experiences by identifying areas of need and guiding the future efforts of this group.

Several recurrent themes emerged as patients and caregivers shared their experiences: (1) delayed IFD diagnosis (reported by 5/6 [83%] of patients), (2) importance of caregivers as surrogate decision-makers and patient advocates, (3) impact of diagnosis and treatment on quality of life (QoL), (4) gratitude for their healthcare provider(s), and (5) strong commitment to improve the experience of future patients. These themes were ubiquitous throughout the summit and repeatedly discussed during subsequent breakout sessions.

To further probe these themes, a follow-up survey was completed by 3 patients. Participants reported typically seeing multiple healthcare providers in different settings before fungal diagnosis, except for the patient diagnosed with COVID-19-associated pulmonary aspergillosis. For 1 patient, several putative diagnoses including lung cancer and gall bladder disease were advanced before the fungal diagnosis was finally obtained.

**Figure 1.**
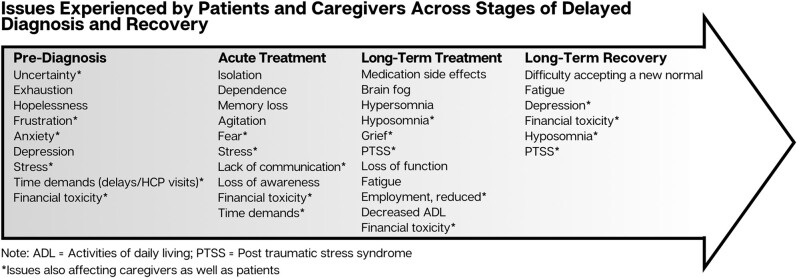
Invasive fungal disease treatment journey with common challenges faced at different stages.

As mentioned previously, cognitive issues, sleep disturbances, and posttraumatic stress syndrome (PTSS) plagued the patients and their caregivers. Moreover, financial toxicity was an issue that emerged, not only from diagnostic delays and unnecessary testing, but also given the length of treatment, hospitalizations, and the inability of either the patient and/or the caregiver to work regularly. For some patients, lingering issues with fatigue and cognitive changes necessitated a career change or retirement. Figure 1 summarizes the reported experiences across the stages of IFD diagnosis, acute therapy, follow-up therapy, and long-term recovery. Because symptoms consistent with PTSS were experienced (and continue to be experienced) in patient and caregiver participants, the recovery process continues for most of them.

### Identifying Unmet Needs and Key Priorities

During general discussions and breakout sessions, unmet needs were explored in terms of educational, research, and advocacy needs. Consensus on key priorities was reached (Table 1).

**Table 1.** Key Mycology Priorities Identified at the Summit

Main Topics	Top Priorities
Education	Web-based information
	What to expect across disease stages and settings
	Material to help caregivers optimize care for loved ones
	Guidance on dealing with long-term effects
	Identifying and getting help for mental health side effects
Research	New diagnostics development
	New drugs that are effective and safe
	Patient-reported outcomes
	Desirability of Outcome Response (DOOR)
	New clinical trial methodologies
	Documenting risks and disease burden
	Fungal isolate banks
Advocacy	Public and provider education about fungal diseases—educating them to “think fungal” at all levels of training
	Reaching primary care for streamlined referrals
	Financial resources, funding
	Frequently asked questions with responses
	List of questions for patients to ask providers
	Peer-to-peer programs for patient/caregiver support
	Creating educational programs for medical students, clinicians

#### Educational Unmet Need and Priorities

Patients and caregivers prioritized web-based opportunities for peer-to-peer interactions to support patients and caregivers who want to talk about and gain insight about their fungal disease experience from others with shared experience. Many of the caregivers (and some patients) tried to research their disease on the internet yet found little to no information. Therefore, they stressed the importance of developing a website that provides vetted information and supports those peer-to-peer interactions. It should define what each fungal disease is and how it is contracted, advise how to speed the time to diagnosis, review optimal treatment (with a review of efficacy and side effects), and provide financial resources. Caregivers also wanted specific guidance on how to best provide care during the different stages of the journey, specifically regarding (1) the diagnostic process, (2) hospitalizations, (3) through treatments and recovery, and (4) while addressing long-term disabilities and psychological impacts. The clinicians requested a resource to hand patients to direct them to the MyCARE site to drive traffic. Side effect trackers could also be a resource that the MyCARE site could include as patient reported outcome instruments are developed.

The clinician participants agreed with these priorities for patients'/caregivers' education and outreach needs. They also emphasized the importance of educating patients about recognizing and working collaboratively with their healthcare providers to manage side effects so patients can get the best from therapy. They also thought patients would benefit from information about prognosis and what to expect, but they emphasized the importance of fostering hope rather than focusing on negative statistics.

The mycology expert and clinician participants also prioritized a role for education to encourage the medical community to “think fungal” and employ effective diagnostics. One key issue that was discussed and supported by the patient stories was the significant lag-time in diagnosis of IFD. The clinicians corroborated this experience, relaying that by the time the patient is hospitalized with an infectious diseases' consultation or referral to infectious diseases by a primary care physician, lag times have occurred that may have dire consequences. The clinicians also agreed that they need more documentation about the patient's experience to inform their treatment practices and communication strategies. This can be difficult to quantify when signs and symptoms may start ambiguously. They acknowledged the importance of making quality fungal education easy to find at initial web searches and expressed concern about patients accessing misinformation from unverified sources of information.

During the mycology expert/clinician breakout session, major deficits in undergraduate medical education were highlighted as a potential reason for shortages in the infectious diseases' specialty and, in particular, mycology. They highlighted that the foundation of mycological education is established in medical school; however, as more demands are placed on the curriculum, there is less time dedicated to fungal diseases. They suggested that the protracted delays of diagnosis in 5 of the 6 patient participants may have been potentially averted by a more robust medical mycological education. Reinforcing these principles of medical mycology in residency training in the primary care specialties would also contribute to improved diagnostic acumen.

#### Research Unmet Needs and Priorities

The mycology experts and clinicians attending the summit were also clinical mycology researchers who emphasized key research priorities for the field. They identified a major unmet need as diagnostics. The group discussed that there is little federal support for advances in clinical mycology diagnostics and emphasized that better and easier-to-use diagnostic options should reduce diagnostic delays. They discussed the need for developing new rapid molecular, immunodiagnostic, and biomarker assays. They also stressed the importance of establishing large fungal isolate banks to support diagnostic development. Similarly, as antifungal resistance and increased frequency of drug-drug interactions emerge as issues, new antifungal drug development is needed. The clinical research participants stressed the need for meaningful incentives from sponsors such as the National Institutes of Health, the Food and Drug Administration, and diagnostics/pharmaceutical companies to support these efforts.

Participants also discussed a need for novel clinical trial designs that include validated patient-important (reported) outcome measures to better define success and failure of antifungal medications. One researcher stated that “Current clinical trial outcomes use ‘mortality’ and ‘complete cure’ as criteria for success; but we must move beyond these.” Another pointed out that the MSGERC Desirability of Outcome Ranking initiative, which uses an innovative approach to considering global benefits and risks of antifungal drugs with patient-important outcomes, is addressing this issue. From an epidemiology standpoint, the participants reached consensus to prioritize updated definitions of disease burden and evidence-based definitions for risk across IFDs. As 1 clinician researcher pointed out: “New science has identified genetic predispositions and concomitant medications such as biologics that can impact fungal disease risk.”

Both the patient/caregiver and mycology expert/clinician groups concurred about these general research priorities. Together, they stressed the importance of documenting the current epidemiology and burden of illness for fungal diseases to provide an evidence base to advocate for additional funding and resources to “think fungus” and “fight fungus.” Collectively, they expressed hope that the MyCARE advocacy initiative can serve as a means to increase patient access for the patient-important outcomes validation and clinical trial recruitment. Overall, there was considerable concurrence between attendee groups on mycology research needs.

#### Advocacy Unmet Needs and Priorities

The patients/caregivers agreed that part of the advocacy effort is education of the public and healthcare community. They want all the people who are involved with mycology—family, friends, healthcare providers, as well as regulators and elected officials—to have a better understanding of the burden of fungal disease on a personal level. This will make it easier to advocate for additional research and access to effective diagnostics and antifungal medications. On a very practical level, patients/caregivers want financial resources, questions to ask their doctor, and peer-to-peer support programs.

The mycology experts and clinician researchers agreed with these priorities but also reiterated that clinicians do not get enough education about fungal disease during their training. In general, there is a need to improve advocacy for the field of clinical mycology. This includes ways to motivate medical students and residents to consider specializing in infectious diseases and medical mycology. Moreover, once a career track in infectious diseases is selected, early-career investigators need mentoring to become committed to academic careers in medical mycology, including mentoring in clinical trials. In addition, the training of all fellows in pediatric and adult infectious diseases training programs should provide core competency in the field of medical mycology to expand the national expertise. The future advocacy and advancement of the field depends on this as the key opinion leaders in mycology begin to retire. MyCARE should have an active role in ensuring the future of mycology.

### Final Priorities

The final session asked the groups to address the highest priorities to support clinical mycology. Patients and caregivers want increased resources and funding for medical mycology, improved diagnostic testing, and meaningful input into patient-centered research. Clinician researchers want to collaborate with advocacy groups and learn more about that process, they too want an increase in patient-centered research and a better understanding of how to use patient important outcomes and real-world evidence in future clinical research in drug and diagnostics development. They also want to understand current epidemiology on risk factors and true impact of IFD in US and global populations.

The groups recognized the importance of bringing fungal disease to the forefront—doing whatever it takes to raise awareness, increase funding, and capture the patient's voice. Two patient advocacy groups: the Henry Schueler Foundation and the Save Our Sick Kids Foundation were introduced by Dr Thomas Walsh, based on his work in mucormycosis and pediatric IFD. Collaborations with those groups are planned. Overall evaluation responses revealed enthusiastic commitment by the patient/caregiver and clinician researcher attendees to continue working with MyCARE and to develop future goals and objectives for its meaningful growth.

## DISCUSSION

This first ever pan-fungal disease patient, caregiver, and mycology expert/clinician summit captured many unmet needs and priorities to advance clinical mycology goals. Stakeholders agreed that much needs to be done to improve the awareness of serious fungal infections and that we need better tools to manage them. Awareness can be enhanced by inclusion of fungal diseases in priority lists (eg, neglected tropical diseases) and emphasizing antifungal resistance by international organizations such as the World Health Organization.

One of the highest priorities identified by the group is accelerating the diagnosis of suspected fungal diseases. Indeed, delayed IFD diagnosis occurred in 83% (5/6) of the patients attending the summit and is consistent with IFD diagnostic delays reported in the literature [22–24]. The deleterious effect of diagnostic delay was not felt in isolation but pervaded other aspects of patient experiences. Because of diagnostic delays, caregivers report the need to request second opinions to find a diagnosis or push for facility transfer for escalation of care. Earlier diagnosis is also an opportunity to improve patient outcomes through earlier treatment initiation.

In patients with IFD, diagnostic and treatment delays are associated with prolonged hospitalization and increased morbidity/mortality [25–28]. Moreover, the financial burden of invasive fungal diseases is skyrocketing. A major component of burden for IFD includes productivity loss (work absenteeism) and premature death [2]. As mentioned previously, more research is needed to better quantify this burden.

Another patient-centered burden is reduced QoL, a problem that remains woefully understudied in patients with IFD and caregivers. Every patient and caregiver at the summit described symptoms/complications that worsened their QoL including “brain fog,” depression, suicidal ideation, worry about familial responsibilities, excessive fatigue, PTSS, persistent nightmares, hyper- and hyposomnia, and others. For other disease states (eg, cancer), researchers have more extensively studied the impact of diagnosis, treatment, and chronic symptoms on QoL measures [29]. The urgent need for more research on QoL in patients with IFD is evidenced by the shared experiences of the patients and caregivers at our summit. Similarly, as in other diseases, the economic burden for caregivers is often an underrecognized negative consequence of IFD. In the United States, 2 of 10 caregivers have to stop working, whereas 4 in 10 reduce their working hours to make time to care for a loved one [30]. Documentation of the caregiver burden in IFD is an area that requires additional investigation.

Patient and caregiver participants reported long-term sequelae frequently seen in post-intensive care syndrome, including exertional dyspnea, vocal changes related to prolonged intubation, reduction of muscle mass and function, memory loss, impaired executive function, and PTSS. The lasting impact of these conditions can greatly affect QoL and require multidisciplinary support and ongoing follow-up care [31, 32]. Symptoms directly attributable to opportunistic IFD are not well-defined in the literature and may be complicated by the presence of underlying illness [25]. Further research in this area would help to establish realistic recovery expectations and enhance the overall understanding of the disease for survivors and their families.

There were limitations of the patient selection that affected the generalizability of the results of this summit. The patients were selected to represent a range of IFDs and because of their commitment to sharing their experiences in a group setting. The patients at this summit were all male, and the majority were insured. For future work, we propose to balance sex, social determinants of health, and other factors when selecting patients. Of note, the male predominance is reasonable, given that male patients are more likely to be diagnosed with IFD than female patients [33]. Nevertheless, the impact of fungal disease on female patients needs to be documented. In addition, we anticipate that the burden of disease will be more profound in patients who are less resourced and we seek more balanced participation in the future.

## CONCLUSION

The inclusion of patients and caregivers in the drug, device, and diagnostics development arena is a priority goal across disease specialties. The MSGERC Desirability of Outcome Ranking working group has been established; new research to study patient reported and important outcomes tools for IFD, such as coccidioidomycoses, and MyCARE international collaborations are under way. This initial pan-fungal disease stakeholder summit was an important step in identifying IFD burden, educational needs, and research goals from a patient-centered approach. Efforts are under way to expand the MyCARE website (fightfungus.org) and recruit patients and caregivers to build a community, assist in designing research protocols, and support peer-to-peer efforts in the United States and internationally. Future summits are planned to maintain momentum on key priority needs and capture the voice of patients in resource-limited settings.

## Notes

***Acknowledgments.*** The authors acknowledge the patients and caregivers who participated in the summit and shared the stories of their journeys. They also thank Alison Marino for reviewing the content from the caregiver perspective.

***Patient consent statement.*** The institutional review board of the Washington University School of Medicine deemed the summit and the reporting of outcomes as exempt from review.

The patients who attended the summit are coauthors on the manuscript and agreed to the content of the publication.

***Financial support*.** The summit was supported by charitable donations or sponsorships from F2G; Astellas Pharma US; Immy; and Melinta Therapeutics United States to Integrita Healthcare Education Foundation. The MSGERC supported this effort in part from funds from the Centers for Disease Control and Prevention United States via a Cooperative Agreement to the University of Alabama at Birmingham, grant #CDC-RFA-CK20-2003.
